# Our Hospital System

**Published:** 1888-05-19

**Authors:** 


### The Hospital

A Weekly Institutional Journal of

**Science, Medicine, Nursing, and Philanthropy.**

*For Hospitals, Asylums, Medical Practitioners, Students, Nurses, Families, Parishes, Congregations, and the Charitable Public.*

Vol. IV. No. 86.] [May 19, 1888.

### Our Hospital System.

There are two courses open to anyone who is called upon to preside at a so-called Festival Dinner on behalf of a charity. He can either content himself with a few platitudes and many details concerning the working of the particular charity the claims of which he has to advocate, or he can take a wider range and raise the tone of the whole proceedings above the common-place level by treating the matter in hand as a question affecting the interests of large masses of the people. It is needless to say Lord Randolph Churchill took the latter course when presiding at St. Mary's Hospital dinner on Saturday last. With that painstaking self-denial which characterises much of Lord Randolph's work, the conscientiousness of which often surprises his friends, Lord Randolph exhaustively studied the hospital system, and what he found to say was not only well said but forcibly put. It is, therefore, to be hoped that the impression this great speech has created will not be allowed to pass away with the echo of his concluding words, but that it will deepen and fructify in the hearts of hospital managers all over the country, and so tend to a more general and wider support being extended to our hospitals everywhere. An evening contemporary has published an article, the writer of which cannot presumably have read this speech, seeing that he takes Lord Randolph to task for setting class against class. All candid readers who peruse the speech published in another part of this issue must see the absurdity of such a charge, whilst they cannot fail to recognise that any sting it possesses is due to the fact that the statements it contains are weighty because they are literally true.

Now what is our hospital system? Lord Randolph declares its main features to be voluntary support combined with free and independent administration. It possesses, indeed, all that enterprise and sound management which attaches to free and independent institutions. But it has also disadvantages which must not be overlooked. These may be summarised by the statement that the income is necessarily uncertain in amount because voluntary; that the managers are often sorely pressed for want of money, amounting on an average to from £100,000 to £200,000 per annum in London alone; that the population is increasing at a rate which makes it difficult to provide adequately and in the best way for the needs of the suffering poor, owing partly to the central position occupied by the majority of the metropolitan hospitals; and that the sites are often so hemmed in by buildings of all kinds that the recovery of the patients is interfered with owing to an absence of air and light. More money is the pressing cry of nearly all the London hospitals, whilst some of the most useful of them, to wit, University College and St. Mary's, are so restricted as to site as to make it absolutely necessary, in the public interest, that the whole area of land of which they at present occupy but a portion shall be secured for the sole use of these institutions without further delay. This latter point is mainly a money question also, and so we are brought face to face with this crying necessity for further funds. How is this money to be obtained? Must the hospitals come on the rates and the voluntary principle of hospital support be admitted to be a failure? Can nothing be done to prevent this great misfortune, which would be rightly held to cover our day and generation with indelible disgrace? Are we less careful for the needs of our suffering neighbours than our forefathers? Has wealth lessened, benevolence diminished, and charity become cold, calculating, or inadequate? Those who know most about such matters would indignantly negative any such assertions. What then is the cause of this want of funds to maintain our hospitals in the fullest efficiency? Undoubtedly, so far as London is concerned—and it is in London mainly that the difficulty is so lamentably apparent—new methods must be found of arousing and fixing public attention, as well as of raising income for our hospitals. Lord Randolph must have had a shrewd suspicion of the facts, or he would not have instinctively put his finger on the two main blots by showing—(1) that annual subscriptions are falling off in proportion to the increase in expenditure; and (2) that the working classes in London do not yet support the metropolitan hospitals to anything like the same extent that the artisans do in the provincial towns. We have not space to-day to go into these points fully, but we commend this most useful speech to the study not only of hospital managers, but of all classes of the community, for all classes are equally interested in the efficiency of all the hospitals. We shall be curious to notice if any of the many millionaires now resident in London, for a portion of each year at any rate, will take the opportunity of immortalising their names which Lord Randolph offers him. We hope that more than one of them may take the trouble to examine an Ordnance map showing the sites of St. Mary's, University College and other hospitals: if they do, we make no doubt those sites will soon be acquired in their entirety, to be held for hospital purposes for generations to come.

One word in conclusion. Lord Randolph Churchill has set an example which it is to be hoped will be generally followed in the near future.

He has not only proved that a festival dinner may be changed from a semi-dreary and tedious task to a highly pleasant and profitable gathering, but he has shown every Metropolitan Member of Parliament that if he is to continue to represent the district of London which at present sends him to the House of Commons, he will have to look into this question of hospital accommodation and to see what can be done to ease the anxiety and sufferings of his poorer constituents. At present much preventible, and therefore needless, suffering is brought upon the poor of London by causes which Lord Randolph has clearly brought out, and any Member who fails to grasp this fact, and to strive to remove the causes which create it, will one day be called sharply to account. We believe, however, that the Metropolitan Members will be quick to recognise what is expected of them, in which case the meeting at the Mansion House on Friday afternoon, June 8th, at half-past two p.m., in support of all the London hospitals, will be attended by a large proportion of the Metropolitan Members. If it is not so attended, the people will be to blame if they do not insist upon an explanation for a display of indifference and callousness which without such evidence we should not have believed possible.

